# Streamlining of a synthetic co‐culture towards an individually controllable one‐pot process for polyhydroxyalkanoate production from light and CO_2_

**DOI:** 10.1002/elsc.202100156

**Published:** 2022-02-15

**Authors:** Franziska Kratzl, Andreas Kremling, Katharina Pflüger‐Grau

**Affiliations:** ^1^ Professorship of Systems Biotechnology Technical University of Munich Garching Germany

**Keywords:** co‐cultultivation, cyanobacteria, polyhydroxyalkanoates, *P* *seudomonas putida*, synthetic consortia

## Abstract

Rationally designed synthetic microbial consortia carry a vast potential for biotechnological applications. The application of such a consortium in a bioprocess, however, requires tight and individual controllability of the involved microbes. Here, we present the streamlining of a co‐cultivation process consisting of *Synechococcus elongatus cscB* and *Pseudomonas putida* for the production of polyhydroxyalkanoates (PHA) from light and CO_2_. First, the process was improved by employing *P. putida cscRABY*, a strain with a higher metabolic activity towards sucrose. Next, the individual controllability of the co‐culture partners was addressed by providing different nitrogen sources, each exclusively available for one strain. By this, the growth rate of the co‐culture partners could be regulated individually, and defined conditions could be set. The molC/molN ratio, a key value for PHA accumulation, was estimated from the experimental data, and the necessary feeding rates to obtain a specific ratio could be predicted. This information was then implemented in the co‐cultivation process, following the concept of a DBTL‐cycle. In total, the streamlining of the process resulted in an increased maximal PHA titer of 393 mg/L and a PHA production rate of 42.1 mg/(L•day).

**Abbreviations**
DBTL: design‐build‐test‐learn
HPLC: high performance liquid chromatography
IPTG: isopropyl‐β‐D‐thiogalactopyranoside
3‐MB: 3‐methyl benzoic acid
OD: optical density
PBR: photobioreactor
PHA: polyhydroxyalkanoates
PHB: polyhydroxybutyrate

## 1 INTRODUCTION

In nature, most microorganisms are found in consortia with other microbes, sometimes consisting of hundreds to thousands of species. These natural consortia form a complex network of versatile interactions and offer many advantages for their inhabitants, such as increased resistance towards chemical or mechanical stresses or the joint conversion of a specific compound by the interplay of the different species. In general, a consortium is equipped with more genes than a single microbe, as such possessing a greater genetic diversity, which enables it to perform complex metabolic tasks. For instance, simpler and less refined substrates can be metabolized with higher efficiency than in monocultures, or more complex compounds can be degraded. Thereby, the exchange of small molecules, metabolites, or other substances allows the microbes to coordinate their activity and enables multistep processes. This collective effort can be understood as division of labour, where microbes specialized in different tasks, work together with their neighbours for the benefit of all [1]. The concept of division of labour has been adopted by metabolic engineering approaches and finds application in the rational design of synthetic consortia for biotechnological applications. Natural microbial consortia are already employed for a long time in the preparation of food or beverages, wastewater treatment, or biogas production. In recent years, however, the idea of rationally designed synthetic consortia became more and more prominent. The rationale behind this is that by choosing suitable microbial partners a well‐defined synthetic co‐culture can be created, where the biosynthetic labour is partitioned between the partners to make a specific bioproduction performance either possible or improve it [2, 3, 4, 5, 6, 7, 8, 9, 10]. The possible advantages of a co‐culture over axenic cultures include the metabolisation of otherwise toxic metabolites, the provision with nutrients not accessible to the monoculture, or the formation of a protected environment by for example, the encapsulation in a biofilm.

One combination of microbes that has gained a lot of attention in recent years is the association of photosynthetic microbes with heterotrophic bacteria [3, 5], in which the phototroph provides the heterotroph with nutrients while the heterotrophic organism prevents the accumulation of photosynthetically produced O_2_ by respiration and in turn supplies the phototroph with additional CO_2_. This approach combines effective CO_2_ fixation with the metabolic and biotechnological versatility of heterotrophic bacteria. Crucial aspects for the design of synthetic phototrophic communities are the selection of the involved strains, the screening for suitable cultivation conditions, and the development of a controlled bioprocess.

A photosynthetic strain that has been employed in various synthetic phototrophic co‐cultivations is the cyanobacterium *Synechococcus elongatus cscB* [11, 12, 13, 14, 15, 16], engineered to excrete sucrose [17]. This strain carries the *cscB* gene, encoding a sucrose/H^+^ symporter from *Escherichia coli* integrated into the chromosome. When exposed to elevated salt concentrations, *S. elongatus* naturally accumulates sucrose as a compatible solute in the cytoplasm to counteract the osmotic pressure. The engineered *S. elongatus cscB* thus secretes sucrose into the environment in conditions of osmotic stress by the activity of the CscB transporter [17]. This sugar then in turn serves as carbon source for the co‐culture partner. As co‐culture partners different microbes have been employed, comprising *Azotobacter vinelandii* [11, 13], *Bacillus subtilis* [16]*, Escherichia coli* [16], *Halomonas boliviensis* [15]*, Pseudomonas putida* [12, 18], and yeast strains [14]. In some cases, these co‐cultures served for the production of polyesters, like polyhydroxybutyrate (PHB) or medium‐chain‐length polyhydroxyalkanoate (mcl‐PHA). PHA is a linear polymer of 3‐hydroxy fatty acids with different chain length, that accumulates under non‐optimal conditions as both, carbon and energy storage compound in certain bacteria, among them *P. putida*. It is naturally accumulated in conditions of carbon overflow and limitation of another nutrient like nitrogen or phosphorus. Due to its thermoplastic, polypropylene‐like properties, it is handled as a valuable substitute for conventional petroleum‐based plastics, as it is biocompatible and biodegradable [19, 20].

**PRACTICAL APPLICATION**

One of the major challenges of our time is the replacement of fossil resources by sustainable alternatives. The co‐cultivation process for the production of polyhydroxyalkanoates (PHA) by the synthetic consortium of a cyanobacterium and a PHA producing heterotrophic bacterium presented in this work has the potential to contribute to the sustainable production of bioplastics. These biopolymers are already used in some applications as an alternative to petroleum‐based plastics. The combination of the cyanobacterial carbohydrates as feedstock together with the biosynthetic potential of *Pseudomonas putida*, an emerging workhorse in Synthetic Biology, opens a wide range of applications beyond the production of PHA. Our vision is to provide a platform process in which cyanobacterial feedstock is used by *P. putida* to produce metabolites ‘*a la carte’*, made possible by the genetic introduction of the respective metabolic pathway(s) in *P. putida*.

Our approach was the association of *S. elongatus* PCC7942 *cscB* and *P. putida* in a one‐pot process for the production of PHA [12]. In order to provide *P. putida* access to the sucrose excreted by *S. elongatus cscB*, it was genetically modified to metabolize sucrose, resulting in *P. putida* EM173 *cscAB* [21]. In this one‐pot process, *S. elongatus cscB* secreted sufficient sucrose to support the growth and PHA accumulation of *P. putida* with a maximal titer of ~150 mg PHA/L [12].

Nevertheless, the process had a couple of shortcomings, which left room for improvement and are addressed in this work here. Thus, a major fraction of the sucrose was left untouched by the *P. putida* variant. This was tackled by employing *P. putida cscRABY*, a strain with a higher metabolic activity towards sucrose [22] than the previously used *P. putida cscAB* [21]. Many other parameters of the process were neither controllable nor measurable. For example, it could not be differentiated between the share of nitrogen metabolized by each co‐culture partner. In consequence, the process conditions chosen were a trade‐off between providing enough nitrogen for the cyanobacterium to grow and produce sucrose, but sufficiently low nitrogen to induce a PHA accumulation regime by nitrogen limitation in *P. putida*. This was addressed by uncoupling the PHA‐production from the presence of nitrate by using a nitrate blind variant of *P. putida cscRABY* carrying a deletion of the *nasT* gene, which could no longer grow with nitrate as nitrogen source [18], but growth on other nitrogen sources, for instance ammonium was not affected.

In summary, here, we will present the stepwise streamlining of a synthetic co‐culture towards a highly controllable process with increased PHA production from light and CO_2_ adopting a design‐build‐test‐learn (DBTL) cycle concept.

## 2 MATERIALS AND METHODS

### 2.1 Bacterial strains and batch cultivation

For the synthetic co‐culture, the photoautotrophic organism *S. elongatus cscB* [17] and two heterotrophic *P. putida* variants *P. putida attTn7::cscRABY* [22] or *P. putida attTn7::cscRABY ΔnasT* [18], respectively, were used. Pre‐cultures of *S. elongatus cscB* were propagated in 100 mL shake flasks with 20 mL of BG‐11^+^ [12] at 120 rpm, 25°C and a constant photon flux density of 20 μmol/(m^2^•s) in an incubation shaker (Multitron Pro from Infors HT, Switzerland), whereby air was the sole source of CO_2_. Pre‐cultures of *P. putida* strains were grown in 5 mL LB‐medium overnight at 30°C and 220 rpm (MaxQ 8000 from Thermo Scientific, USA). Subsequently, an aliquot of 100 μL was transferred to 5 ml BG11^+^ medium supplemented with 1–3 g/L sucrose and, in the case of the *P. putida cscRABY ΔnasT*, 1.0 g/L urea was added. With these pre‐cultures, the main cultures were inoculated in a defined ratio of 1:1000 [% v/v] in 250 ml shake flasks, and the BG11^+^ medium was additionally supplemented with 150 mM NaCl. The main cultures were cultivated under the same conditions as the pre‐cultures.

### 2.2 Photobioreactor (PBR) cultivations

Litre‐scale cultivations of the synthetic co‐culture consisting of *S. elongatus* *cscB* and *P. putida* *att*Tn7::*cscRABY ΔnasT* or *P. putida* *att*Tn7::*cscRABY*, respectively, were performed in a Labfors 5 Lux flat panel airlift PBR (Infors AG, Switzerland) at a photon flux density of 240 μmol/(m^2^•s). The pH was adjusted to 7.4 with 1 M NaOH, except for conditions of non‐N‐limited growth of *S. elongatus* *cscB*, where 1 M HNO_3_ was used. An airflow of 1.96 L/min enriched with 2 % CO_2_ was used as a carbon supply. The BG‐11^+^ cultivation medium was supplemented with 150 mM NaCl resulting in a final process volume of 1.8 L. For inoculation, 10–20 ml of a stationary *S. elongatus* *cscB* culture was used to reach a start OD_750_ of 0.05–0.1.

Nitrate limited processes were supplemented with batch nitrate (NaNO_3_) of 50 mg/L and, subsequently, nitrate supply was ensured by a HNO_3_—feed (74‐110 mg/(L•day) for the process with *P. putida* *cscRABY* and 105 mg/(L•day) for the processes with *P. putida* *cscRABY ΔnasT*. The feed was controlled gravimetrically. Nitrate unlimited processes used BG11^+^ with a standard sodium nitrate concentration of 1.5 g/L. After growth of the cyanobacterium up to an optical density (OD_750_) of approximately 0.5, 0.1 mM isopropyl‐β‐D‐thiogalactopyranoside (IPTG) was added to induce expression of the *cscB* symporter and thus sucrose export. Simultaneously, *P. putida* pre‐cultures were prepared (see batch cultivation section). After 1–2 days, the co‐culture was started by inoculation with *P. putida* cells. For the process with *P. putida* *cscRABY*, batch urea of 36 mg/L was added. For the processes with *P. putida* *cscRABY ΔnasT*, different urea feeding rates were adjusted (see Table 1 and Table 2). Sterile polypropylene glycol (antifoam) was added to the bioreactor when needed. A sample of 5–10 ml was taken once or twice a day via the super safe sampler (Infors HT, Switzerland) for measuring the optical density, the cell count, and the analysis of carbohydrates (sucrose, fructose, and glucose), PHA, and nitrogen (urea and/or nitrate). Thereby, the process volume was kept constant by removing approximately an equal volume for sampling as added with the nitrate feed.

### 2.3 Optical density and cell counting

The optical density of cyanobacterium *S. elongatus cscB* and, with progressing process of the co‐culture, was measured with a photometer (BioSpectrometer; Eppendorf, Germany) at a wavelength of 750 nm. Samples of the culture were diluted with BG11^+^ medium, if necessary (light path =  1 cm). If not stated differently, the numbers given are the means of three independent replicates and the standard deviation for growth rates was derived from the error of the simple linear regression. For analysis of the cell count by flow cytometry (Cytoflex, Beckman coulter, USA), samples were diluted with filtered NaCl (Ø = 0.22 μm, 8.5 g/L) to reach an abort rate of less than 5 % at a flow rate of 10 μL/s and 200,000 fixed events per μL. The flow cytometer used was equipped with two lasers (488 and 638 nm) which allowed for the measurements of fluorescence in the range of 488–‐585 nm and 640–780 nm. The fluorescence in the higher emission range was used to identify the cyanobacterial population. The fluorescent dye RH414 (N‐(3‐triethylammoniumpropyl)‐4‐(4‐4‐(diethylamino) phenyl) butadienyl) pyridinium dibromide) (rh414, 3 μM, AAT Bioquest, USA) was used to distinguish the *P. putida* cells from the background noise by a fluorescent shift at 488–585 nm emission wavelengths.

### 2.4 Measurement of urea, nitrate and sugar concentrations

Samples of 1 mL volume were centrifuged for 5 min at 13,000 rpm (Eppendorf centrifuge 5418; Germany), cell pellets were frozen at –80°C, and supernatants were stored at ‐20°C. The urea concentration of the culture supernatant was determined using the urea/ammonia‐assay Kit of Megazyme (NEOGEN, Megazyme, USA), which is based on the enzymatic oxidation of NADH to NAD^+^ at a wavelength of 340 nm and was scaled down for a microplate reader. For nitrate determination, the NO_2_/NO_3_—assay from ENZO (Enzo life science, USA) was used and the user manual was strictly followed. Carbohydrate concentration (sucrose, glucose, and fructose) was determined by high‐performance liquid chromatography (HPLC) using an Agilent machine (Agilent 1100 series; Agilent Technologies, USA). For separation, a sugar shodex SH1011 column (Shodex, Shawa Denko Europe, Japan) and a mobile phase of 0.5 mmol/L H_2_SO_4_ at 30°C and a flow of 0.45 mL/min were used. All sample supernatants were filtered (Ø = 0.22) and supplemented 1:1 with 0.2 g/L Na_2_‐EDTA·dihydrate prior to the measurements.

### 2.5 PHA recovery and determination

Cell pellets were stored at ‐80°C until PHA quantification was performed. For this, the pellets were freeze‐dried at 0.08 mbar and –60°C (Alpha 1–2LDplus from Martin Christ, Germany). Then, samples were supplemented with 300–400 μL chloroform and a 1:1 ratio a mixture of 1‐propanol and 37 % HCL (80 % / 20 %, v/v) was added. 0.1–0.5 g/L polyhydroxy butyrate (PHB) and 0.1–0.5 g/L 3‐methyl benzoic acid (3‐MB) solved in chloroform were added as internal standards. The test tubes were closed tightly, the content was mixed, and the samples were incubated for at least 10 h at 80°C with occasional mixing. After incubation, the pellet was completely dissolved. Samples were washed with 600 μL H_2_0 (Retch MM 200 ball mill 25 Hz, 3 min, Retch GmbH Germany) and centrifuged for 5 min at 13 000 rpm (Eppendorf centrifuge 5418) for phase separation. The organic phase was dried with NaSO_4_ and neutralized with NaCO_3_. After this, 100 μL were transferred into GC vials. As external standard 3‐hydroxyoctanoate and 3‐hydroxydecanoate were used and treated the same way as the samples. The samples were separated with a ZB‐WAX column (Phenomenex, USA, length: 30 m, ID 0.32, film 0.25 μm) and measured with a flame ionisation detector (245°C). The injection volume was 1 μL with a split ratio of 1:10 and the injector temperature was set to 240°C (AOC‐20i autoinjector SHIMADZU, Japan). Hydrogen gas was used as carrier gas at a flow rate of 3 mL/min. The different PHA‐monomers were separated by applying a temperature gradient, starting at 80°C for 1 min, afterwards linearly increasing by 5°C every minute, stopping at 240°C with a holding time of 5 min.

## 3 RESULTS AND DISCUSSION

### 3.1 Co‐cultivation of *S. elongatus cscB* with *P. putida cscRABY*, a variant with increased sucrose metabolizing activity

The first step towards an improvement of the synthetic co‐culture of *S. elongatus cscB* and *P. putida* was to employ the recently constructed strain *P. putida cscRABY* [22] as the heterotrophic partner in the co‐cultivation process. In contrast to *P. putida cscAB* used in the previously published co‐culture study [12], the ‘new’ co‐culture partner strain possesses an increased capability for sucrose consumption. This was achieved by chromosomal integration of the complete sucrose utilisation operon *cscRABY* derived from *Pseudomonas protegens* Pf‐5. It encodes not only the permease CscB and the invertase CscA, but also the regulator CscR and the porin CscY. Especially the latter one was shown to be crucial for a stable sucrose consumption phenotype [22].

**FIGURE 1:**
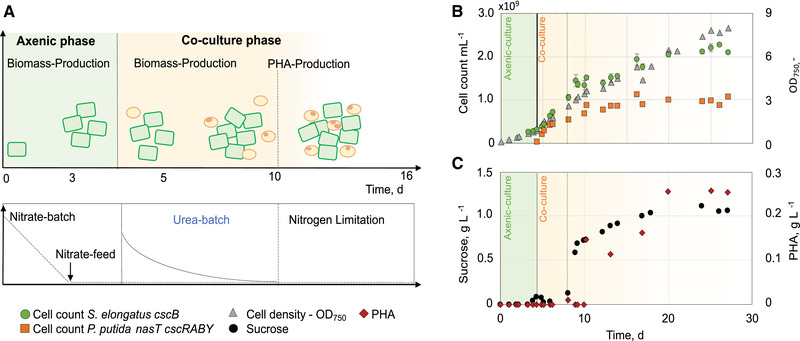
Co‐culture of *S. elongatus* *cscB* with *P. putida* *cscRABY* with nitrate limitation in 1.8 L scale (Process 1). The overall process was divided into two phases: Axenic‐culture of *S. elongatus* *cscB* with a nitrate batch of 50 mg/L and a nitrate feed of 74 mg/(L•day) (green area). Co‐culture phase started with inoculation (vertical line) of *P. putida* *cscRABY* and supplementation of a urea batch of 36 mg/L. Nitrate feed was adjusted to 110 mg/(L•day) (yellow area). (A) Graphical overview of the co‐culture process. (B) Growth of the co‐culture partners represented in cell count and OD_750_. Numbers given are the mean of technical triplicates and the error bars represent the standard deviation. (C) Sucrose and PHA concentration determined in single measurements. Process conditions: BG11^+^ supplemented with 150 mM NaCL at 30°C and pH 7.4 (not controlled); Aeration: 1.96 NL/min air with 2 % CO_2_; Illumination: constant at 240 μmol/(m^2^•s); Induction of *cscB* expression with 0.1 mM IPTG

We separated the co‐cultivation process into two phases, a first axenic culture phase, in which only *S. elongatus cscB* was cultivated, and the subsequent co‐cultivation phase, which was started by the addition of *P. putida cscRABY* to the process vessel (Figure 1). During the whole process, the optical density was determined at 750 nm, reflecting the overall cell density of both strains. Additionally, sucrose, urea, and nitrate concentrations, as well as the PHA accumulation were analysed and the cell counts of each strain were monitored by flow cytometry. Therefore, samples were stained with the fluorescent dye RH414, which accumulates in the phospholipid membrane and allows quantification of the two co‐culture populations as well as differentiation (Figure S1).

In order to have more control over the process, we used a nitrogen‐free medium, which allowed for the regulation of microbial growth by adjusting the nitrogen availability. The first axenic phase with an initial amount of 50 mg/L nitrate served as biomass production and adaptation phase. After depletion of the batch nitrate (after ∼45 h), marked by a drop in the pO_2_‐% (Figure S2), a constant nitrate feed of 74 mg/(L•day) was set, resulting in linear growth. During the whole process, nitrate was at very low levels around the detection limit of the assay (0.04 mg/L). After an OD_750_ of 0.6 was reached, sucrose secretion was switched on by induction of the expression of the heterologous CscB symporter in *S. elongatus cscB* [17], and cells were grown another 30 h to increase the extracellular sucrose concentration (Figure 1).

The co‐culture phase was started with the inoculation of *P. putida cscRABY*, together with the addition of urea in a concentration of 36 mg/L. We chose urea as an amendment to specifically boost the growth of *P. putida cscRABY*, as it can be exclusively metabolized by this organism and not by the cyanobacterium *S. elongatus cscB* (Figure S3). Simultaneous to the inoculation, the nitrate feed was raised to 110 mg/(L•day). The higher availability of nitrate resulted in an increase in the growth of *S. elongatus cscB* to 23.8 × 10^7^ ± 1.62 × 10^7^ cell/(mL•day). Still, all nitrate was readily consumed by the microorganisms, suggesting the feed was high enough for *S. elongatus cscB* for the accumulation and excretion of sucrose and at the same time low enough to provide a N‐limited regime for *P. putida* necessary for PHA production. The urea provided upon inoculation with *P. putida cscRABY* was metabolized completely, when *P. putida* reached a cell count of 0.5 × 10^9^ per ml (OD_600_ approximately 0.6), and nitrate served as the sole nitrogen source shared by both populations, resulting in linear growth of *P. putida cscRABY* of 5.52 × 10^7^ ± 1.26 × 10^7^ cells/(mL•day) (calculated from process time 150 to 330 h). Simultaneously, sucrose started to accumulate in the supernatant, reaching a maximum titer of 1.1 g/L. These conditions of carbon overflow and nitrogen limitation are favourable for the accumulation of the natural produced storage compound PHA in *P. putida* [23, 24]. Therefore, with the depletion of the urea *P. putida cscRABY* entered the PHA production phase. The intracellular PHA was quantified after extraction from the cells by gas chromatography. In the overall process, a maximum titer of 256 ± 2 mg/L was reached, which corresponds to an average of 0.12 pg PHA/cell (compare Table 5). A production rate of PHA of 9 ± 2.13 mg/(L•day) (10.2–26 days) was reached. The low rate is a result of the strong nitrogen limitation of the cells and the long PHA‐production phase of ∼14.5 days. The composition of the heteropolymer did not change in comparison to previous studies [12], with 3‐hydroxydecanoic acid being the most abundant monomer (Table S1). However, as *P. putida* participates in the nitrate feed supplied for *S. elongatus cscB*, even though probably to a low extent, this presents an unknown variable in the overall process.

### 3.2 Growth control of *P. putida cscRABY ΔnasT* in the synthetic co‐cultivation process though individualisation of the nitrogen sources

To achieve optimal conditions for PHA accumulation in a reliable and reproducible process, it is necessary to set a defined molC/molN ratio. To this end, the nitrogen source for each co‐culture partners was individualized by employing *P. putida cscRABY ΔnasT*, a strain no longer able to metabolize nitrate due to the deletion of the NasT response regulator protein of the two‐component system NasS/NasT [18]. Consequently, nitrate was exclusively available for *S. elongatus cscB*. To enable and control the growth of *P. putida cscRABY ΔnasT* and set the PHA producing condition, urea was used as a nitrogen source for this organism.

**FIGURE 2:**
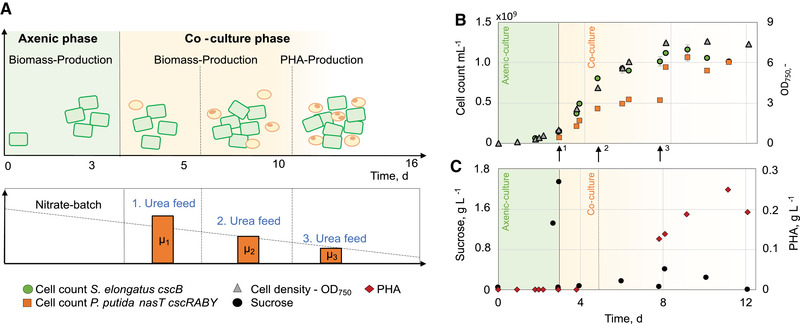
Co‐culture of *S. elongatus* *cscB* with *P. putida* *cscRABY ΔnasT* with urea‐feed in 1.8 L scale (Process 2). The overall process was divided into three phases: Axenic culture of *S. elongatus* *cscB* with non‐limiting nitrate supply (green area). Co‐culture phase (orange area) started with the inoculation (vertical line) of *P. putida* *cscRABY ΔnasT* and comprised three different urea‐feeding rates indicated by arrows: 13.3 mg/(L•day) (${↑}^{1}$) 6.7 mg/(L•day) (${↑}^{2}$) and 2.2 mg/(L•day) (${↑}^{3}$). PHA‐production phase initiated by nitrogen limitation (dashed lines). (A) Graphical overview of the process with three different urea feeds; μ_1_‐ μ_3_ indicate the growth rate of *P. putida* *cscRABY ΔnasT* with the respective urea feed. (B) Growth of the co‐culture partners represented in cell count and OD_750_. Numbers given are the mean of technical triplicates and the error bars represent the standard deviation. (C) Sucrose and PHA concentration. PHA data represent the results of single or duplicate measurements. Process conditions: BG11+ supplemented with 150 mM NaCL at 30°C and pH 7.4 (controlled with HNO_3_); Aeration: 1.96 NL/min air with 2 % CO_2_; Illumination: constant at 240 μmol/(m^ 2^•s); Induction of *cscB* expression with 0.1 mM IPTG

In order to show the individual tunability of the growth of *P. putida cscRABY ΔnasT* by urea, three different feeding rates were applied during the co‐cultivation phase (Figure 2), while *S. elongatus cscB* was cultivated without nitrogen limitation.

As in the process described before, a first axenic phase took place in which, upon induction of sucrose secretion, up to 1.6± 0.13 g/L of sucrose was detectable in the supernatant. During the process *S. elongatus* showed a sigmoidal growth behaviour with a growth rate of 12.3 × 10^7^ ± 3.5 × 10^7^ cells/(ml^•^day) in the linear section (2.5–5 days). The change from exponential to linear growth can most likely be attributed to limitation of light. After 8 days of process time growth of *S. elongatus* ceased completely, assumingly due to light limitation because of self‐shading effects.

Upon inoculation *with P. putida cscRABY ΔnasT* (after 70 h), the sucrose accumulated in the medium in the first axenic phase, was readily metabolized by this organism. The urea feeding rate of 13.3 mg/(L•day) resulted in linear growth of *P. putida cscRABY ΔnasT* with a rate of around 2 × 10^11^ cells/(ml•day) (Table 1). Throughout this first phase of the co‐culture, *P. putida cscRABY ΔnasT* experienced both, carbon and nitrogen limitation as sucrose levels stayed at very low levels. In total, 36 mg/L of urea were supplied in this first feeding phase. This amount equals the amount of urea that was served as batch in the process described above with *P. putida cscRABY*. After this biomass generation phase, a cell count of 0.5 × 10^9^ cells/ml was reached, which compared very well to the one reached in the process described above after the urea batch was consumed. Now, the PHA production phase was initiated by generating a stronger nitrogen limitation due to reduction of the urea feeding rate by two to 6.7 mg/(L•day), which likewise resulted in half of the growth rate of *P. putida cscRABY ΔnasT* of around 1.1 × 10^11^ cells/(mL•day). To test the effect of a further reduction of the urea supply on the growth of *P. putida cscRABY ΔnasT*, in a third phase 2.2 mg/(L•day) were fed. Except for the value of the cell number of *P. putida cscRABY ΔnasT* right upon the change in the feeding rate, the cell number stayed constant at around 1 × 10^9^ cells per ml. From the data it is not clear, whether this value was a result of the strong limitation, had a physiological origin, or was due to technical reasons.

**TABLE 1:** Urea feeding rate and specific growth rate of *P. putida* *cscRABY ΔnasT* (Process 2)

	Urea feed [mg/(L•day)]	Growth rate^a^ [cells/(mL•day)]	Nitrogen concentration
Feeding‐rate 1	13.3	2.0 × 10^11^ ± 1.6 × 10^10^	High
Feeding‐rate 2	6.7	1.1 × 10^11^ ± 5.8 × 10^10^	Medium
Feeding rate 3	2.2	5.8 × 10^9^ ± 3.9 × 10^10^	Low

^a^ Non‐specific growth rate.

Analysis of the PHA concentration revealed, that the polymer was accumulated primarily in the second and third feeding phase. In these two phases growth was reduced to an extent that allowed sucrose to accumulate in the medium, which created a regime of carbon overflow and nitrogen limitation. In the overall process a maximum titer of 209 ± 33 mg PHA/L was reached with a maximal PHA production rate of 33  ±4.4 mg/(L•day) (compare Table 5). The maximum titer compares well to the process described before, however, due to the considerably reduced process time, the PHA production rate was about twice as high. At the very end of the process (after around 12 days), when no sucrose was detectable anymore, PHA levels slightly decreased again, presumably due to metabolisation by *P. putida cscRABY ΔnasT*.

In this co‐cultivation approach, we were able to individualize the nitrogen source by using a combination of strains and nitrogen source that makes it exclusively available for only one of the partners. This allowed us to set the growth rate of the heterotrophic organism without affecting the growth of the cyanobacterium, a prerequisite for individually tuning the performance of the strains.

### 3.3 Differential control of each co‐culture partner through defined conditions

**FIGURE 3:**
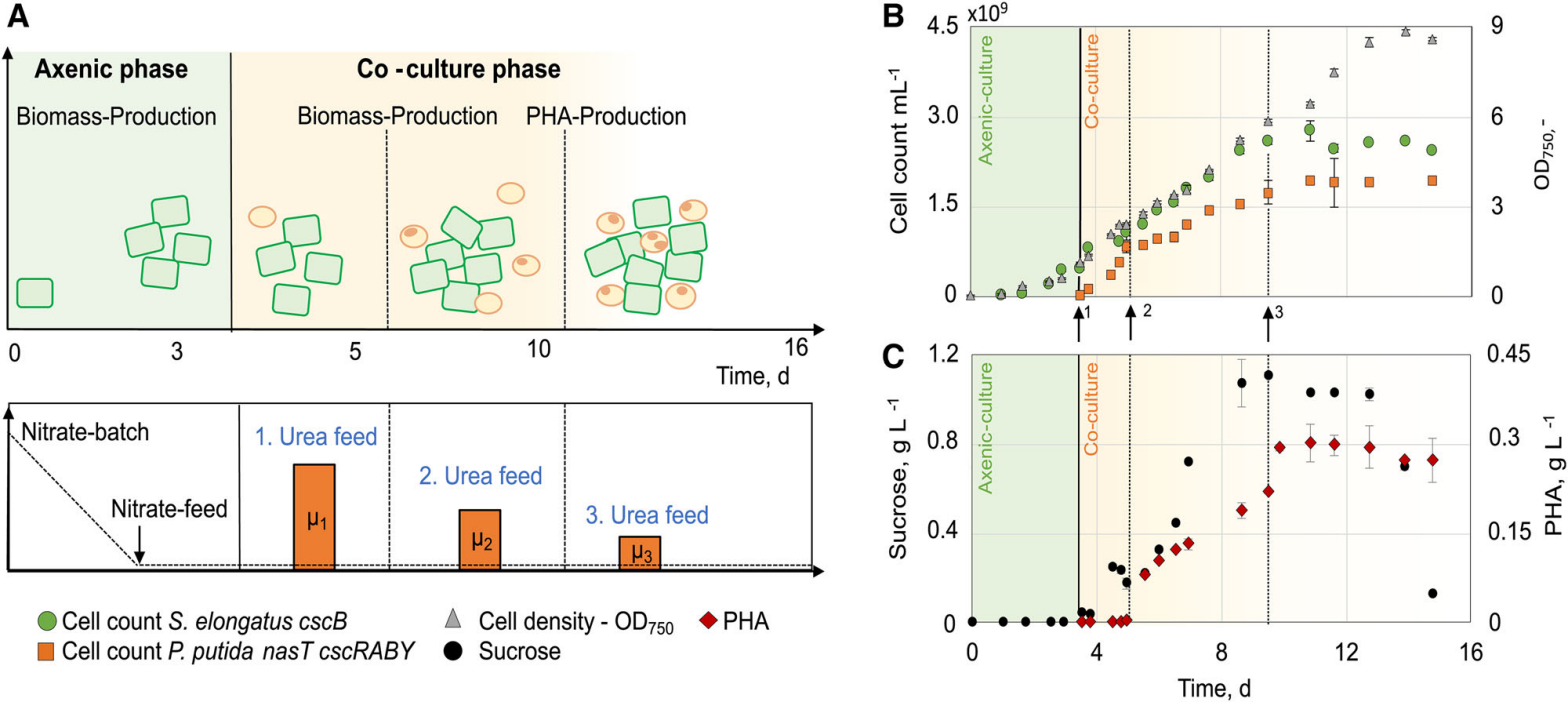
Co‐culture of *S. elongatus* *cscB* with *P. putida* *cscRABY ΔnasT* with N‐limitation for both co‐culture partners in a 1.8 L scale process (Process 3). The overall process was divided into three phases: Axenic‐culture of *S. elongatus* *cscB* with a nitrate batch of 50 mg/L and a nitrate feed of 105 mg/(L•day) (green area). Co‐culture phase (yellow area) started with inoculation (vertical line) of *P. putida* *cscRABY ΔnasT* and comprised three different urea feed rates indicated by arrows: 40 mg/(L•day) (${↑}^{1}$), 20 mg/(L•day) (${↑}^{2}$), and 2.2 mg/(L•day) (${↑}^{3}$). PHA‐production phase initiated by nitrogen limitation (dashed lines). (A) Graphical overview of the process with three different urea feeds; μ_1_‐ μ_3_ indicate the growth rates of *P. putida* *cscRABY ΔnasT* with the respective urea feed. (B) Growth of the co‐culture partners represented in cell count and OD_750_. Numbers given are the mean of technical triplicates and the error bars represent the standard deviation. (C) Sucrose and PHA concentration. PHA measurements were performed in duplicates. Process conditions: BG11^+^ supplemented with 150 mM NaCL at 30°C and pH 7.4 (not controlled); Aeration: 1.96 NL/min air with 2 % CO_2_; Illumination: constant at 240 μmol/(m^2^•s); Induction of *cscB* expression with 0.1 mM IPTG

Based on the approach described above, we set out to further enhance the controllability of the co‐cultivation with the aim of increasing the amount of PHA produced. Therefore, it was necessary not only to have a tight and precise control over the growth of the heterotrophic organism but also to manipulate the growth of the cyanobacterium. A logical consequence was to combine both processes described above by applying two nitrogen feeds, a nitrate feed for *S. elongatus cscB* and a urea‐feed for *P. putida cscRABY ΔnasT* (Figure 3). This differential feeding strategy allowed us to specifically set and control the growth rates of both partner strains individually. In order to improve the PHA production, we aimed to increase the volumetric PHA yield of the overall process by increasing the cell number of *P. putida cscRABY ΔnasT*. This strategy was chosen, as the PHA content per cell already was in the range of around 30 % of the cellular dry weight, which is described as an upper limit in *P. putida* strains grown on non‐PHA related carbon sources [25]. Therefore, the sucrose production time was prolonged by limiting the growth of *S. elongatus cscB* by the availability of the nitrogen source. This should result in a higher overall sucrose yield and, in consequence, for higher cell numbers of *P. putida cscRABY ΔnasT*.

As in all processes shown here, first an axenic culture of *S. elongatus cscB* was performed for initial biomass and sucrose production, again, as in the first approach with an initial nitrate batch in a concentration of 50 mg/L (Figure 3A). This axenic culture phase compared well to the first approach with the nitrate being consumed after around 45 h, marked by a drop in the pO_2_‐%. In contrast to the first process, a nitrate feed of 105 mg/(L•day) was chosen. This higher availability of nitrate is reflected by the higher growth rate of *S. elongatus cscB* of around 33.4 × 10^7^ cell/(ml•day). Furthermore, the final cell count reached was around 2.6 × 10^9^ cells/ml, which is more than twice as high as observed in the previous processes (Figure 3B).

For *P. putida cscRABY ΔnasT*, three different urea‐feeding rates were applied as in the previous process (Figure 3A and Table 2). These were chosen to provide different ratios of carbon to nitrogen, as PHA accumulation is known to be dependent on the carbon to nitrogen (molC/molN) ratio [26]. The first feeding rate was set to 40 mg/(L•day), which is three times higher than one of the previous process (Process 2). This resulted in a growth rate of about 4.9 × 10^8^ cells/(ml•day) and a final cell count of 8.5 × 10^8^ cells/ml at the end of this phase (Figure 3B). In this phase, a slight accumulation of sucrose of around 0.2 g/L was already detectable, suggesting that growth was still limited by the nitrogen source (Figure 3C). The second and third feeding rates were adjusted to 20 and 2.2 mg/(L•day) in order to provide a regime for PHA production with medium and high nitrogen limitation. In total, 2.3 times more urea was fed than in the previous process. The second feeding rate, which was half of the first feeding rate, resulted in a growth rate of around 2.3 × 10^8^ cells/(ml•day), which is about half of the growth rate observed in the first feeding phase. With the third feeding rate, which was a factor of around 9 lower than the second, the growth rate consequently was also a factor of around 9 lower. Thus, we could predictably set the growth rate of *P. putida cscRABY ΔnasT* by adjusting the urea feeding rate, while the growth of *S. elongatus cscB* was not influenced.

**TABLE 2:** Urea feeding rate and specific growth rate of *P. putida* *cscRABY ΔnasT* in the process with individual N‐feeds (Process 3)

	Urea feed [mg/(L•day)]	Growth rate^a^ [cells/(mL/•day)]	molC/molN ratio
Feeding‐rate 1	40	4.9 × 10^8^ ± 0.8 × 10^8^	low
Feeding‐rate 2	20	2.3 × 10^8^ ± 0.2 × 10^8^	medium
Feeding rate 3	2.2	2.7 × 10^7^ ±0.2 × 10^8^	high

^a^ Non‐specific growth rate

In terms of PHA accumulation, we could also observe an influence of the adjusted urea feeding rate (Figure 3C). In the first feeding phase, where the lowest molC/molN ratio was present, no PHA accumulation could be detected. However, in the second feeding phase, where less urea was supplied, and therefore a higher molC/molN ratio is expected, PHA started to accumulate with a rate of 33 ± 1.1 mg/(L•day) (Table 5). Sucrose accumulated in the medium, clearly indicating that growth of *P. putida cscRABY ΔnasT* was limited by the nitrogen source. In the third feeding phase, where the molC/molN ratio is expected to be even higher by a strong reduction of the urea concentration in the feed, no further increase in the amount of PHA accumulated could be observed, except for the sample point directly after the change to the third feeding rate. The maximum titer reached in this approach was 292 ± 10 mg/(L•day) (Table 5). The sucrose concentration stayed at a constant level of around 1 g/L until day 13 when it started to decrease.

On the 11th day, there was a shift in the co‐culture behaviour. Both organisms stopped growing as the cell counts ceased, but the optical density kept increasing for the next 3 days (Figure 3B). This increase in the OD cannot be attributed to a further increase in the PHA concentration, as cell size and PHA production correlate with each other (see Figure S4). It might rather be the consequence of other morphological or metabolic changes. In fact, this phenomenon was also observed in the first co‐cultivation approach (see Figure 1).

### 3.4 Estimation of the settled carbon to nitrogen (molC/molN) ratio

A molar ratio of the concentration of carbon to nitrogen (molC/molN) in the medium of approximately 26 was described to be optimal for PHA accumulation in *Pseudomonas* species [27, 28]. In order to bring the data obtained in the process described above into context, it was necessary to get a good estimation of the molC/molN ratio applied. Whereas the concentration of nitrogen is known, as it is defined by the urea feed, the factual concentration of carbon in the medium cannot be measured directly, as part of the sucrose secreted by *S. elongatus cscB* is consumed directly by *P. putida cscRABY ΔnasT* and converted to cell mass, PHA and used for cell maintenance. Therefore, first, a good estimation of the amount of sucrose secreted by *S. elongatus cscB* had to be found.

The sucrose concentration in the bioreactor $\frac{dc_{suc}}{dt}\;\left(g\;L^{-1}\;d^{-1}\right)$ is described through the differential Equation (1). It considers, the sucrose section by *S. elongatus cscB* and the sucrose uptake of *P.  putida cscRABY ΔnasT*. This apparent sucrose accumulation rate ($r_{acc\_suc}$) can be described as:

(1) $$\frac{dc_{suc}}{dt}=r_{acc\_suc}=r_{\sec\_suc}-q_{up\_suc\;}\cdot \;c_{P.putida}$$

whereby $c_{P.putida\;}\left(g\;L^{-1}\right)$ represents the cell dry weight of *P. putida*. The sucrose specific uptake rate is given by $q_{up\_suc}\;\left(h^{-1}\right)$ and the sucrose secretion rate by $r_{\sec\_suc\;}\left(g\;L^{-1}\;h^{-1}\right)$. After transposition of Equation (1) to the unknown rate, Equation (2) follows:

(2) $$r_{\sec\_suc}=r_{acc\_suc}+q_{up\_suc\;}\cdot \;c_{P.putida}$$

The sucrose specific uptake rate by *P. putida cscRABY ΔnasT* can be defined as:

(3) $$q_{up\_suc}=\frac{\;\mu_{P.putida}}{Y_{Xsuc}}+q_{PHA}$$

With $\mu_{P.\;putida}$ $\left(h^{-1}\right)$ being the specific growth rate and $Y_{Xsuc}\;\left(g\;g^{-1}\right)$ the biomass yield of *P. putida cscRABY ΔnasT* from sucrose. The specific product formation rate of PHA is represented by $q_{PHA}\;\left(h^{-1}\right)$. This term can be insert in formula (2) resulting in Equation (4):

(4) $$r_{\sec\_suc}=r_{acc\_suc}+\left(\frac{\mu_{P.putida}}{Y_{Xsuc}}+q_{PHA}\right)\cdot \;c_{P.putida}$$

The growth rate ($r_{P.putida\;=\;}\mu_{P.putida}\cdot \;c_{P.putida}$) and the PHA production rate ($r_{PHA\;=\;}q_{PHA}\cdot \;c_{P.putida}$) could be extracted from simple linear regression of the experimental data in the respective time period (see Figure S5). The same procedure was used for calculating the apparent sucrose accumulation rate ($r_{acc\_suc}$). The biomass yield from sucrose ($Y_{Xsuc})$ of *P. putida cscRABY* was determined previously to be 0.23 g/g ± 0.02 and was considered constant [22].

As a last step, the sucrose secretion rate ($r_{sec\_suc}$) can be transformed into a molar carbon secretion rate ($r_{mol_{c}}$) which is described by Equation (5):

(5) $$\begin{array}{ccc} r_{\mathrm{mo}l_{c}}\left[\mathrm{mol}\;h^{-1}\right] & = & \left(r_{\mathrm{acc}\_\mathrm{suc}}+\frac{r_{P.\mathrm{puti}\mathrm{da}}}{Y_{\mathrm{Xsuc}}}\right)\;\cdot \frac{12\cdot V_{R}}{M_{\mathrm{suc}}}\\ & & +\;r_{\mathrm{PHA},\mathrm{mol}}\cdot 9.63 \end{array}$$

whereby $V_{R}\;\left(L\right)$ represents the reactor volume, $M_{suc\;}\left(g\;mol^{-1}\right)$ the molar mass of sucrose and $r_{PHA,mol}\;\left(mol\;h^{-1}\right)$ the molar PHA production rate. The number 9.63 represents the average number of carbon atoms in the heteropolymer mcl‐PHA.

Taking the molar carbon secretion rate ($r_{mol_{c}}$) and the urea feed ($r_{mol_{N}}$) into account, the molC/molN ratio can be calculated with Equation (6):

(6) $$\frac{C}{N}\left[-\right]=\frac{r_{mol_{c}}}{r_{mol_{N}}}$$

Therefore, by applying Equation (6) the molC/molN ratio of the different urea‐feeding phases could be calculated (Table 3 and see Figure S6). The ratio in the first feeding phase was 20.1, as a high urea feed was applied. In the second phase, the molC/molN ratio was calculated to be around 31. Both ratios are close to 25, which is described to be optimal for PHA accumulation, however, no PHA accumulation was detectable with the ratio of 20.1 (see Figure 3C). The molC/molN ratio of the third phase was very high, as the urea feed was extremely low, but could not be calculated from the data, as no further increase in the sucrose concentration was measured.

**TABLE 3:** Estimated molC/molN ratio for *P. putida* *cscRABY ΔnasT* (Process 3)

	Estimated molC/molN ratio
**Feeding‐rate I**	20.1
**Feeding‐rate II**	31
**Feeding rate III**	n.a.

Abbreviation: n.a., not applicable.

### 3.5 Improvement of the process by setting the optimal molC/molN ratio for PHA accumulation

**FIGURE 4:**
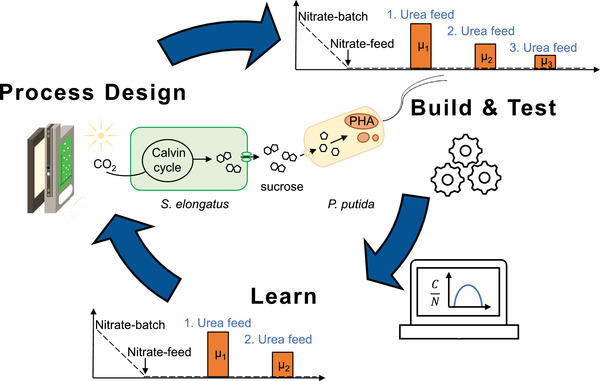
Design Build Test Learn cycle for co‐culture engineering. The key aspects of each phase are presented. The cycle starts with the process design (DESIGN), which is then applied (BUILD) and tested (TEST). From the data derived from that phase, by means of a mathematical approach (LEARN), an improved design of the process was predicted

To improve the overall process even more, we adopted the Design‐Build‐Test‐Learn (DBTL) engineering strategy from Synthetic Biology [29] (Figure 4). In general, the DESIGN phase is followed by the BUILD and TEST phase, where the experimental data is obtained. In the LEARN phase, information is extracted and processed, which allows the prediction of an improved process design for the next round.

**FIGURE 5:**
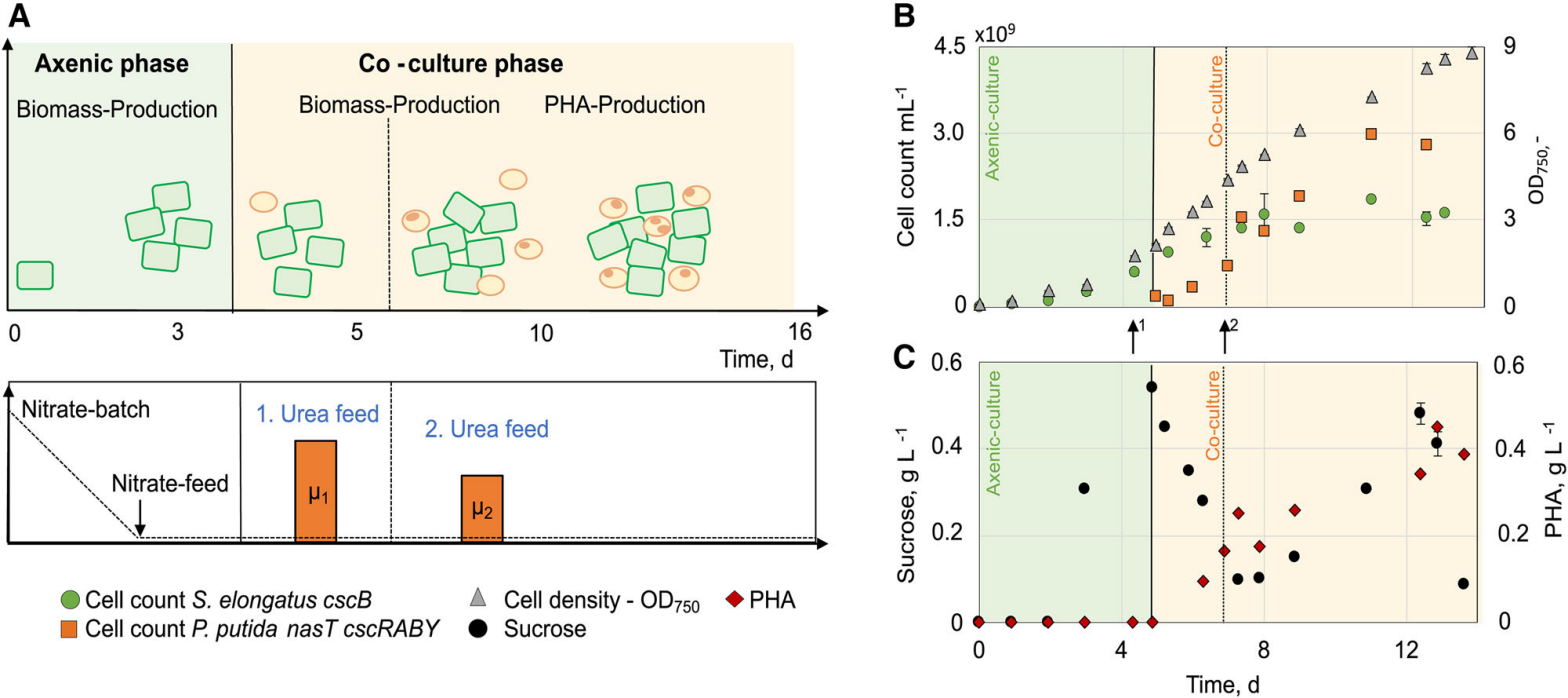
Co‐culture of *S. elongatus* *cscB* with *P. putida* *cscRABY ΔnasT* with an optimized molC/molN ratio in a 1.8 L scale process (Process 4). The overall process was divided into two phases: Axenic‐culture of *S. elongatus* *cscB* with a nitrate batch of 50 mg/L and a nitrate feed of 105 mg/(L•day) (green area). Co‐culture phase (yellow area) started with inoculation (vertical line) of *P. putida* *cscRABY ΔnasT*. Two different urea feed rates were applied, indicated through arrows: 40 mg/(L•day) (${↑}^{1}$) and 25 mg/(L•day) (${↑}^{2}$). PHA‐production phase through nitrogen limitation is indicated with a dashed line. (A) Graphical overview of the process. μ_1_ and μ_2_ represent the growth rates of *P. putida* *cscRABY ΔnasT* with the respective urea feed. (B) Growth of the co‐culture partners represented in cell count and OD_750_. Numbers given are the mean of technical triplicates and the error bars represent the standard deviation. (C) Sucrose and PHA concentration. Sucrose and PHA measurements were performed in single or double measurements. Process conditions: BG11^+^ supplemented with 150 mM NaCl at 30°C and pH 7.4 (not controlled); Aeration: 1.96 NL/min air with 2 % CO_2_; Illumination: constant at 240 μmol/(m^2^•s); Induction of *cscB* expression with 0.1 mM IPTG

Here, we used the information extracted from the experimental data of the previous processes to predict the necessary urea feeding rate to obtain an optimal molC/molN ratio for PHA production (LEARN). Thus, the DESIGN phase can be understood as a process design phase to predict the configuration of the feeding strategy to adjust the apparent molC/molN ratio in the bioreactor and concomitantly the accumulation of PHA. The process was designed basically as above, however, after the axenic phase, only two distinct co‐cultivation phases were planned, one for biomass formation of *P. putida* *cscRABY ΔnasT* and a subsequent phase for PHA accumulation (Figure 5A). The conditions for the phototrophic co‐culture partner *S. elongatus cscB* were chosen to be identical as described above. The PHA accumulation phase was designed in a way to reach a molC/molN of 26. By assuming that the molar carbon rate, which is equivalent to the sucrose secretion rate of *S. elongatus cscB*, is constant, an increase of the nitrogen feed should lower the molC/molN ratio (compare Table S3 and S4). Therefore, the feeding rate of the PHA accumulation phase should be increased by 25 % to reduce the molC/molN ratio from 31 to 26. To achieve this, the urea‐feeding rate was set to 25 mg/(L•day).

The axenic phase compared well to the ones of the processes before and sucrose was readily accumulated and reached a maximum of 0.54 g/L (Figure 5B). The first co‐culture phase served, as in the prior processes, as biomass production phase of *P. putida* *cscRABY ΔnasT* with the same urea feed being supplied as in the process above (Table 4), resulting in a similar growth rate of about 5.1 × 10^8^ cells/(ml•day). In the PHA production phase the growth rate of *P. putida* *cscRABY ΔnasT* decreased due to N‐limitation, however, was still faster than in Process 3, as more nitrogen was available due to the decreased molC/molN ratio (Table 4). This faster growth led to a higher sucrose consumption rate, which was reflected by lower sucrose levels in the medium compared to Process 3 (Figure 5C). Taking the experimental data of the designed process into account, the molC/molN ratio was calculated to be 25.8 (Table 4), which is very close to the desired one of 26. The PHA accumulation rate was determined to be 42.1 mg/L. However, PHA accumulation started already in the biomass production phase suggesting that the heterotrophic cells were stressed and perceived signals of limitation. Furthermore, the ratio in the first phase could not be estimated due to decreasing sucrose concentrations (see Figure S7). The cell count of *P. putida* *cscRABY ΔnasT* was higher than in the other processes since more carbon was available for biomass production and maintenance. This is also reflected by the higher maximum PHA titer of 393 ± 53 mg/L. At the end of the process, nearly all sucrose was consumed by *P. putida* *cscRABY ΔnasT*, indicating that with this process the cell count of *P. putida* *cscRABY ΔnasT* was already in the range of the maximum possible, as there is a natural limitation due to the amount of sucrose produced by *S. elongatus* *cscB* [12, 17].

**TABLE 4:** Urea feeding rate, specific growth rate and C/N ratio of *P. putida* *cscRABY ΔnasT* in the designed process (Process 4)

	Urea feed [mg/(L•day)]	Growth rate^a^ [cells/(ml•day)]	molC/molN ratio
Feeding‐rate I	40	5.1 × 10^8^ ± 1.45	—
Feeding‐rate II	25	3.5 × 10^8^ ± 1.15	25.8

^a^ Non‐specific growth rate

A comparison of the processes in respect to PHA accumulation is shown in Table 5. With the designed Process 4, the maximum PHA titer could be clearly increased, though mainly due to a higher biomass of *P. putida* *cscRABY ΔnasT*. The volumetric PHA production rate was also slightly increased in the designed Process 4 compared to the other processes, whereas the PHA content per cell remained constant in Process 4 compared to Process 3. Thus, the adjustment of the molC/molN ratio towards the optimal ratio, did not result in an improvement of the PHA content per cell, suggesting that the conditions chosen in Process 3 were already in the range of the optimum.

**TABLE 5:** Overview over accumulated PHA in the processes

	Process 1	Process 2	Process 3	Process 4
PHA maximum titer [mg/L]^a^	256 ± 2	209 ± 33	292 ± 10	393 ± 53
PHA [mg/(L•day)]	9 ± 2.13	33 ±4.4	33 ±1.1	42.1 ± 5.8
PHA per cell [pg/cell]^a^	0.12 ±0.0006	0.21 ± 0.02	0.16 ±0.006	0.16 ± 0.012

Abbreviation: PHA, polyhydroxyalkanoate.

^a^ Average of saturation phase.

The relationship between the molC/molN ratio and the PHA production in *P. putida* strains is well known, though the regulation seems to be more complex than simply checking substrate availability [30]. Thus, an improvement in the molC/molN ratio cannot be linearly transformed to an increased PHA content of the cell, as other regulatory mechanisms play a role. In conclusion, since on the cellular level a physiological optimum already seemed to have been reached in the used strains, room for further improvement of the PHA titer lies on either reaching higher cell counts due to other operational conditions or by further genetic engineering of the *P. putida* strain. To this end, already some attempts have been made by other groups to increase PHA accumulation from the structurally non‐related carbon source glucose [31, 32]. However, these results cannot be transferred to *P. putida cscRABY and P. putida cscRABY ΔnasT* directly, as the uptake and the central carbon metabolism is altered in these strains due to the introduction of the *cscRABY* operon.

## 4 CONCLUDING REMARKS

In this work, a stepwise streamlining towards higher controllability of the co‐culture process for PHA production from light and CO_2_ was presented. The improvement took place on genetic level as well as on the process operation level. The first achievement was the individualisation of the control over both species, by employing more suited *P. putida* strains. By using defined feeding rates of individual nitrogen sources growth rates could be predictably set. This ability to control the growth rates individually and independently from each other is of importance, especially if specific conditions need to be adjusted for improving the process performance. In the case of PHA production, it is advantageous to have a defined molC/molN ratio. Thus, by taking into account the growth and PHA accumulation of *P. putida cscRABY ΔnasT* under nitrogen limiting conditions, we could get a good estimation of the excretion rate of sucrose of *S. elongatus cscB*. This allowed us to determine the molC/molN ratio, which was present in the process, and correlate it with the PHA productivity. With this achievement, we were able to calculate the urea feed necessary to achieve the molC/molN ratio, described as optimal for PHA production [27, 28]. This concept of design‐build‐test‐learn (DBTL) is an engineering principle adopted from Synthetic Biology, where the design phase is the conceptual representation of the system, the build phase is the actual physical sample, which will then be tested to obtain the data necessary to learn and predict parameter, that then in turn can be applied in the next cycle [33]. This iterative DBTL cycle requires both dry and wet lab approaches, as presented in this work here, though that the shares depend on the problem to solve. With this approach, we adjusted the process phases and designed the co‐cultivation process in a way to reach the molC/molN ratio described as optimal for PHA production. Though an increase of 35 % in the maximum PHA titer was observed, no further increase in the PHA content could be achieved on the single‐cell level, suggesting that a physiological optimum was already reached. Further improvement of the synthetic consortium, therefore, relies on other strategies, as working with high cell densities or employing other cyanobacterial strains with higher sucrose production rates, as for instance *S. elongatus* UTEX 2973 [34].

## CONFLICT OF INTEREST

The authors declare no conflict of interest.

## Supporting information

SUPPORTING INFORMATION

## Data Availability

The data that supports the findings of this study are available in the supplementary material of this article.
