# Supplementary material for: Streamlining of a synthetic co‐culture towards an individually controllable one‐pot process for polyhydroxyalkanoate production from light and CO2
Source: Eng Life Sci. 2022 Feb 15;23(1):e2100156. doi: 10.1002/elsc.202100156 (PMC9815089; doi:10.1002/elsc.202100156)
Supplement: Supplementary file 1 — SUPPORTING INFORMATION [file ELSC-23-e2100156-s001.pdf]

Supporting Information

Streamlining of a synthetic co-culture towards an individually controllable one-pot process for polyhydroxyalkanoate (PHA) production from light and CO_2

*Franziska Kratzl, Andreas Kremling, Katharina Pflüger-Grau**

Professorship of Systems Biotechnology, Technical University of Munich, 85748 Garching (Germany)

* For correspondence: Katharina Pflüger-Grau, Professorship of Systems Biotechnology, Technical University of Munich, Boltzmannstr. 15, 85748 Garching (Germany), Tel.: +49 89 289 15765; Fax.: +49 89 289 15766, email: k.pflueger-grau@tum.de

Table S1: Distribution of chain length of PHA monomers for each process. Mean and standard deviation were taken from three independent PHA samples.

Chain length	6	8	10	12 & 12:1
**Massfraction Process 1, (%)**	2.4 $\pm$ 0.93	23.1 $\pm$ 2.7	62.2 $\pm$ 1.9	12.2 $\pm$ 0.7
**Massfraction Proocess 2, (%)**	2.4 $\pm$ 0.35	19.6 $\pm$ 2.2	64.3 $\pm$ 1.0	13.7 $\pm$ 1.5
**Massfraction Process 3, (%)**	2.4 $\pm$ 0.31	19.9 $\pm$ 2.0	65.3 $\pm$ 5.1	12.4 $\pm$ 7.0
**Massfraction Process 4, (%)**	2.9 $\pm$ 0.60	24.1 $\pm$ 6.5	60.1 $\pm$ 9.1	12.8 $\pm$ 9.1

Table S2: Applied urea feed and calculated growth rate of Process 3.

	Urea feed [mg L^{-1} day^{-1}]	Growth rate [cells mL^{-1} day^{-1}]
**Feeding-rate 1**	40	$4.90 \times 10^8 \pm 0.76 \times 10^8$
**Feeding-rate 2**	20	$2.30 \times 10^8 \pm 0.19 \times 10^8$
**Feeding rate 3**	2.2	$2.67 \times 10^7 \pm 0.20 \times 10^8$

Table S3: Applied urea feeds and calculated carbon rates in Process 3.

	Urea feed [mmol day^{-1}]	Carbon rate [mmol day^{-1}]
**Feeding-rate 1**	2.4	50 $\pm$ 3.6
**Feeding-rate 2**	1.2	38 $\pm$ 1.4
**Feeding rate 3**	0.13	-

Table S4: Applied feeding rates and calculated carbon rates in Process 4.

	Urea feed [mmol day^{-1}]	Carbon rate [mmol day^{-1}]
**Feeding-rate 1**	2.4	-
**Feeding rate 2**	1.5	38 $\pm$ 9.7

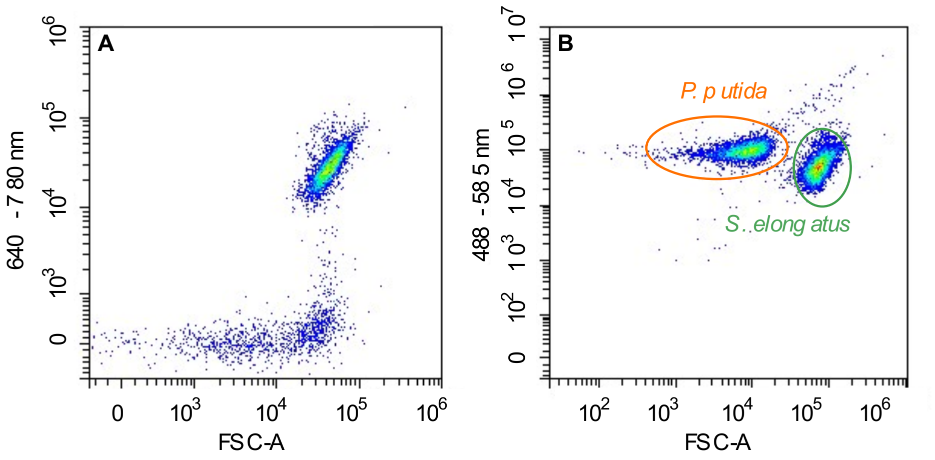

Figure S1: Distinct quantification of both strains by flow cytometry. The flow cytometer used was equipped with two lasers (488 nm and 638 nm). (**A**) The fluorescence emission of chlorophyll could be detected in the filter 640 – 780 nm and allowed hereby for the identification of the cyanobacterial population of the co-culture. (**B**) The fluorescent dye RH414 ((N-(3-Triethylammoniumpropyl)-4-(4-4-(Diethylamino) phenyl) Butadienyl) Pyridinium Dibromide)) was used to distinguish the *P. putida* cells from the background noise by a fluorescent shift at 488 – 585 nm emission wavelength. After gating for cells, the distinction as seen in **B** was achieved.

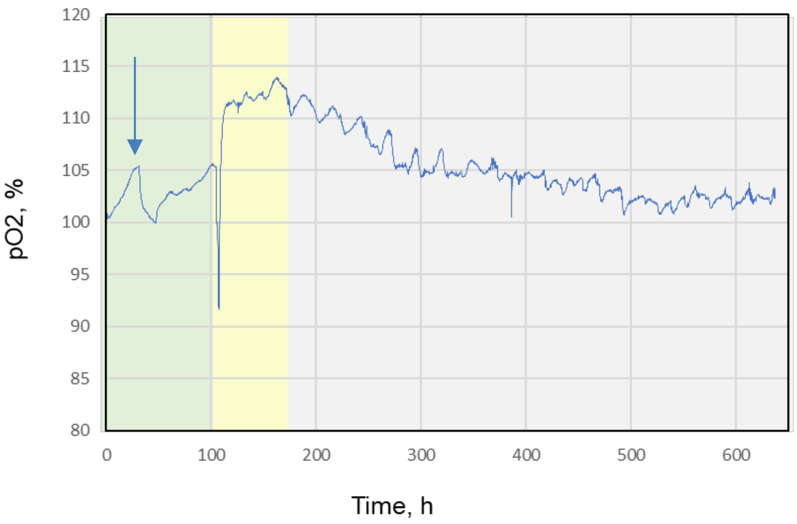

Figure S2: Exemplary recorded pO_2 data from Process 1. Drop in pO_2 was used as signal for complete metabolization of batch nitrate of 50 mg L^{-1} (marked with the arrow).

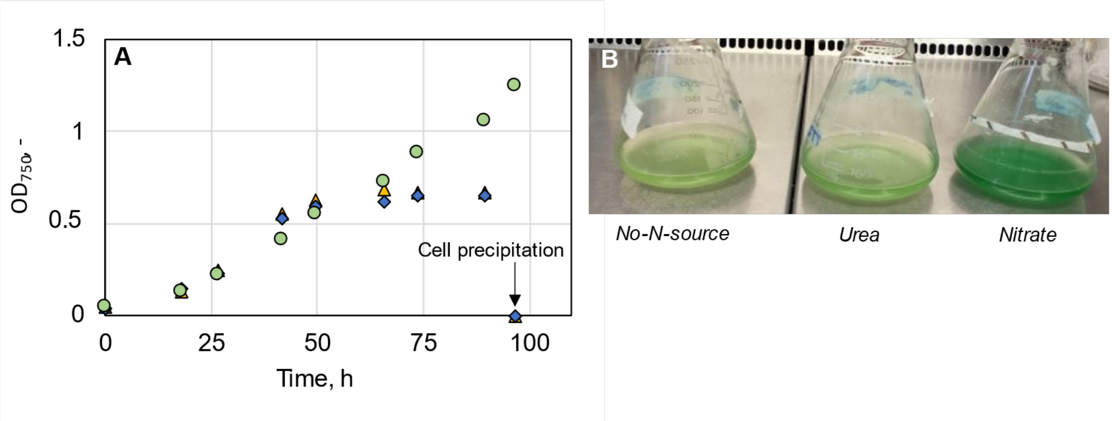

Figure S3: (A) Growth of *S. elongatus cscB* determined with different nitrogen sources. Experimental conditions: 25 mL BG11$^+$ medium with nitrate (green dots), urea (1 g L^{-1}) (blue diamonds) or without nitrogen source (yellow triangles) at 120 rpm, 30 °C with constant illumination at 20 µmol m^{-2} s^{-1} and no extra CO_2 supply. (**B)** Chlorosis observed after 50 h in cultures without nitrogen source or urea as sole nitrogen source, respectively.

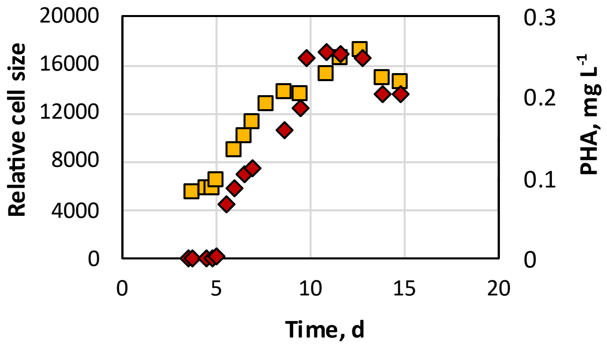

Figure S4: Correlation of relative cell size of *P. putida cscRABY ΔnasT* (in orange squares) and PHA concentration (in red diamonds) (data from in Process 3). The relative cell size was received from FSC-A data of cell count measurements with the flow cytometer. An increase in relative cell size was observed while cells were accumulating PHA.

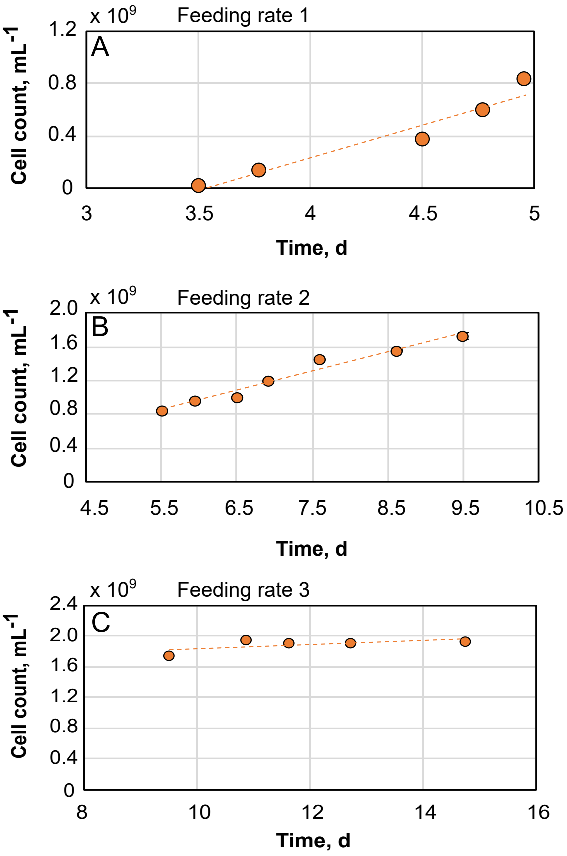

Figure S5: Cell count over time of *P. putida cscRABY ΔnasT* displayed in the sections used for simple linear regression to calculate the growth rate of *P. putida cscRABY ΔnasT* in Process 3. (**A)** Section for feeding-rate 1 (3.5 – 5 d), (**B)** for feeding-rate 2 (5.5 – 9.5 d) and (**C)** for feeding-rate 3 (9.5 – 14.7 d). These sections were also used for the calculation of the heterotrophic biomass to estimate the C/N ratio.

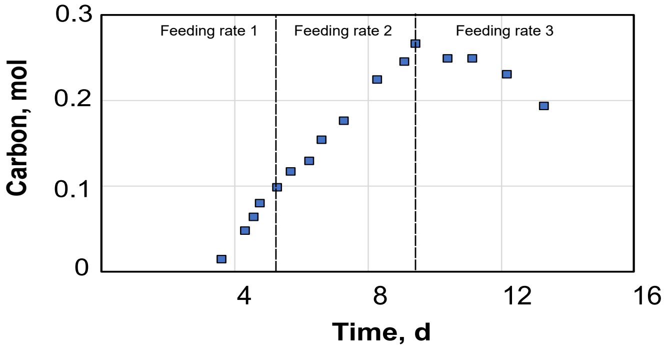

Figure S6: Molar carbon concentration over time in Process 3, calculated from the biomass of *P. putida cscRABY ΔnasT*, the sucrose concentration in the bioreactor, and the PHA accumulated in the heterotrophic co-culture cells. The dashed lines represent the switch of the urea-feeding rates (1-3) and form the boundaries for simple linear regression to calculate the molar carbon rates for each feeding rate, which are shown in Table 3. For feeding rate 3 the carbon rate could not be determined due to the declining overall carbon concentration.

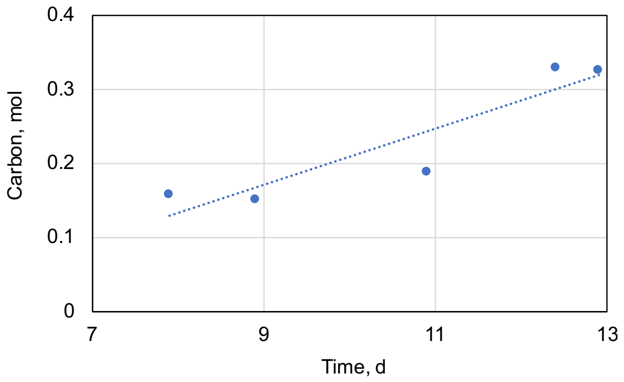

Figure S7: Molar carbon concentration over time for the PHA production phase in Process 4, calculated from biomass of *P. putida cscRABY ΔnasT*, the sucrose concentration in the bioreactor and the PHA accumulated in the heterotrophic co-culture cells. The C/N ratio was estimated from the data marked by the dashed line and are displayed in Table 4.
